# A Postbiotic Consisting of Heat-Treated Lactobacilli Has a Bifidogenic Effect in Pure Culture and in Human Fermented Fecal Communities

**DOI:** 10.1128/AEM.02459-20

**Published:** 2021-03-26

**Authors:** Alicja K. Warda, Adam G. Clooney, Feargal Ryan, Pedro H. de Almeida Bettio, Giulio Di Benedetto, Reynolds P. Ross, Colin Hill

**Affiliations:** a APC Microbiome Ireland, University College Cork, Cork, Ireland; b School of Microbiology, University College Cork, Cork, Ireland; University of Naples Federico II

**Keywords:** *Bifidobacterium*, postbiotic, heat-killed bacteria, health, *Lactobacillus*, microbiome, pharmabiotics, bifidobacteria

## Abstract

The gut microbiota has a significant impact on host health. Dietary interventions using probiotics, prebiotics, and postbiotics have the potential to alter microbiota composition and function. Other therapeutic interventions, such as antibiotics and fecal microbiota transplantation, have also been shown to significantly alter the microbiota and its metabolites. Supplementation of a fecal fermentation model of the human gut with a postbiotic product, Lactobacillus LB, led to changes in microbiome composition (i.e., increase in beneficial bifidobacteria) and associated metabolic changes (i.e., increased acid production). Lactobacillus LB is a heat-treated preparation of cellular biomass and a fermentate generated by Limosilactobacillus fermentum CNCM MA65/4E-1b (formerly known as Lactobacillus fermentum CNCM MA65/4E-1b) and Lactobacillus delbrueckii subsp. *delbrueckii* CNCM MA65/4E-2z, medically relevant strains used to produce antidiarrheal preparations. In pure culture, Lactobacillus LB also stimulates the growth of a range of bifidobacterial species and strains. Lactobacillus LB-like preparations generated using other *Lactobacillaceae*, including commercially available probiotic bacteria, did not have the same impact on a model strain (Bifidobacterium longum subsp. *infantis* ATCC 15697). This bifidogenic activity is heat and enzyme stable and cannot be attributed to lactose, which is a major constituent of Lactobacillus LB. *L*. *fermentum* CNCM MA65/4E-1b is largely responsible for the observed activity, and there is a clear role for compounds smaller than 1 kDa.

**IMPORTANCE** In general, disruptions to the gut microbiota are associated with multiple disorders in humans. The presence of high levels of *Bifidobacterium* spp. in the human gut is commonly considered beneficial. Bifidobacteria can be supplemented in the diet (as probiotics), or those bifidobacteria already present in the gut can be stimulated by the consumption of prebiotics such as inulin. We demonstrate that Lactobacillus LB (a product consisting of two heat-killed lactic acid bacteria and their metabolites) can stimulate the growth of bifidobacteria in human fermented fecal communities and in pure culture. Given the heat treatment applied during the production process, there is no risk of the lactic acid bacteria colonizing (or causing bacteremia in) vulnerable consumers (infants, the immunocompromised, etc.). Lactobacillus LB has the potential to affect human health by selectively promoting the growth of beneficial bacteria.

## Abstract (precis)

In general, disruptions to the gut microbiota are associated with multiple disorders in humans. The presence of high levels of *Bifidobacterium* spp. in the human gut is commonly considered beneficial.

## INTRODUCTION

Gut microbiota composition can play an important role in host health status (1–5). In particular, microbiota disruption has been linked to diarrhea (6), irritable bowel syndrome (IBS) (7), obesity (8, 9), allergies (10), and behavioral and developmental disorders, including autism (3, 11). Altered levels of microbial metabolites have also been associated with many conditions, including depression (12), colorectal cancer (13), cardiovascular disease, obesity, and type 2 diabetes (14). Strategies designed to influence microbiota composition include the ingestion of probiotics (live bacteria providing a health benefit [15]), prebiotics (“a substrate that is selectively utilized by host microorganisms conferring a health benefit” [16]), and synbiotics (a combination of selected substrates and live bacteria that provide a health benefit) (17). More drastic, but less predictable, approaches to microbiota modulation include supplementation with antimicrobials (such as antibiotics [18–20] or bacteriocins [21, 22]) or fecal microbiota transfer (FMT). Recently, heat-killed preparations of microorganisms and/or their preformed metabolites are gaining interest (23–25). Heat-killed bacteria that have demonstrated health benefits have fallen into the definition of postbiotic, a “preparation of inanimate microorganisms and/or their components that confers a health benefit on the target host” (23). Postbiotics have several benefits compared to the use of preparations containing live bacteria (26–29). Notably, nonviable preparations have the potential to be used in immunocompromised individuals without fear of infection, in combination with antimicrobials (without risk of losing activity due to antimicrobial coadministration), and in less developed regions with restricted access to stable storage conditions.

The beneficial effects of bacteria and their metabolites are exemplified by the bifidobacteria. These are anaerobic, Gram-positive bacteria often found in the human gastrointestinal tract. Bifidobacteria produce the short-chain fatty acids (SCFA) propionate and acetate (30) as well as other organic acids, antimicrobial peptides, and quorum-sensing inhibitors (31) previously shown to be valuable to the host (30, 32). Generally, higher levels of bifidobacteria are associated with beneficial effects, including decreased levels of endotoxins in the gut, decreased intestinal permeability, inhibition of enteropathogens, reduction of rotavirus infection, decreased rates of bacterial translocation, and improvement of metabolic parameters such as ameliorated insulin sensitivity and increased high-density lipoprotein (HDL) cholesterol levels (29, 33, 34). At the same time, decreased numbers of bifidobacteria are associated with several disorders, including antibiotic-associated diarrhea, IBS, inflammatory bowel disease (IBD), obesity, allergies, and regressive autism (33).

The stimulation of bifidobacterial levels has been proposed as a strategy to prevent and/or reduce the severity of many disorders and improve quality of life. Bifidobacterial probiotics have been shown to improve symptoms of lactose intolerance, antibiotic-associated diarrhea, IBS, and IBD (31). Stimulation of bifidobacteria can also be achieved by consumption of prebiotics such as inulin, arabinoxylans, galactooligosaccharides (GOS), and fructooligosaccharides (FOS). Therefore, effective and safe stimulation of *Bifidobacterium* levels, particularly intrinsic bifidobacteria (35), seems a valid strategy to prevent and/or reduce the extent of many disorders and improve quality of life.

In a previous study, we showed that prolonged consumption (exceeding 3 weeks) of Lactobacillus LB, a heat-treated fermentate generated by two *Lactobacillaceae* strains, Limosilactobacillus fermentum CNCM MA65/4E-1b (previously Lactobacillus fermentum CNCM MA65/4E-1b [36]) and Lactobacillus delbrueckii CNCM MA65/4E-2z, impacted mouse microbiota, affected animal behavior (27), and reduced the impact of pathogen-induced colitis (26). Lactobacillus LB is a proprietary product produced (and supplied) by Adare Pharmaceuticals SAS and contains the same active pharmaceutical ingredient (API) as Lacteol, an approved antidiarrheal medication (37). The most significant nonbacterial components of Lactobacillus LB are lactic acid, which is generated during initial fermentation by *L. fermentum* CNCM MA65/4E-1b and *L. delbrueckii* CNCM MA65/4E-2z, and lactose, which is added postproduction to facilitate the drying process. Considering the substantial differences in microbiota between mice and humans as well as the generally beneficial role of *Bifidobacterium* spp. in humans, we examined the effect of Lactobacillus LB in a fecal gut fermentation model inoculated with a human standardized fecal slurry (so-called frozen standardized inoculum, or FSI).

## RESULTS

### Microbiological changes in the presence of Lactobacillus LB during 24 h of fecal fermentation in an *in vitro* distal colon model.

**FIG 1:**
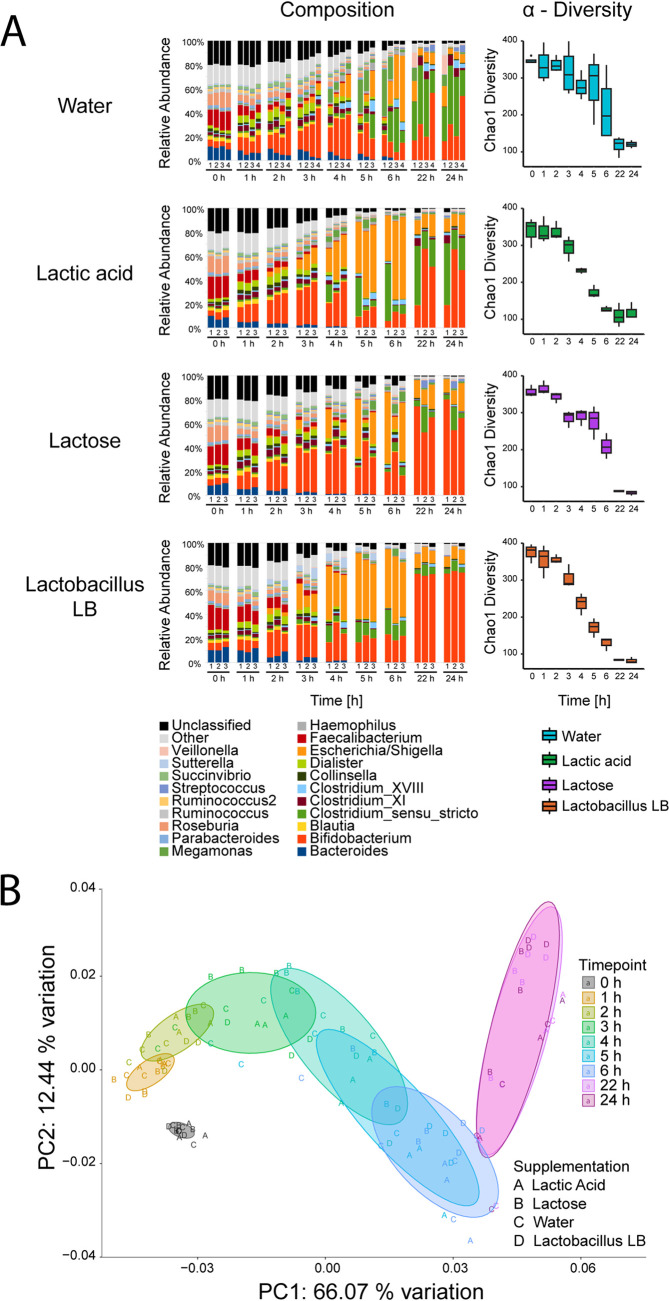
Microbiome analysis during the 24-h fecal fermentation. Vessels were supplemented with water, lactic acid, lactose, or Lactobacillus LB. Samples were collected at the start (0 h) and after 1, 2, 3, 4, 5, 6, 22, and 24 h of fermentation. (A) Composition and α-diversity changes. Left, composition changes at genus level. Each bar represents a sample. The experiment was performed in triplicate (1 to 3) or quadruplicate (1 to 4). For simplicity, all genera with average abundances below 1% were grouped. Right, α-diversity changes measured by Chao1 index. Each bar represents the average for samples taken at each time point. (B) β-Diversity of changes over the 24-h fecal fermentation. Each letter represents sample taken at the start (0; gray) and after 1 (yellow), 2 (light green), 3 (green), 4 (marine), 5 (light blue), 6 (dark blue), 22 (light purple), and 24 (dark purple) h of fermentation. Vessels were supplemented with lactic acid (A), lactose (B), water (C), or Lactobacillus LB (D). The experiment was performed at least in triplicate. Data represent weighted UniFrac values.

A distal colon model was used to investigate the effect of a postbiotic, Lactobacillus LB, on human microbiota. This model utilizes a pH-controlled batch fermentation system inoculated with human frozen standardized inoculum (FSI). Four separate interventions were performed, i.e., water, lactic acid, lactose, and Lactobacillus LB. The lactic acid and lactose concentrations chosen matched the level of both compounds in the Lactobacillus LB preparation (see Table S1 in the supplemental material). At the start of the fermentation in all experimental vessels (Fig. 1A), we observed a high diversity (with 92 genera detected) in the relative abundance at the genus level, as expected from the human gut microbiome. In time, the composition gradually changed in all vessels, with the slowest changes taking place in the water vessel and the fastest in the lactic acid and Lactobacillus LB vessels (Fig. 1A). After an initial increase in the relative abundance of *Escherichia*/*Shigella*, an increase in the relative abundance of *Bifidobacterium* occurred in the Lactobacillus LB vessel and, possibly, to a lesser extent in water and lactic acid vessels (described below) (Table 1, Table S6). Analysis at the ribosomal sequence variant (RSV) level allowed for analysis of more specific taxonomic differences (Table 1, Table S6). The abundance of 26 RSVs was increased (in at least one time point), while 33 RSVs were reduced (in at least one time point), in Lactobacillus LB vessels compared to the water vessel. At the same time, the abundance of only 3 and 14 RSVs changed in at least one time point in the lactic acid and lactose vessels, respectively, compared to the water vessel. Six of the RSVs that increased in the Lactobacillus LB vessel (at 6 and/or 24 h compared to water) could be assigned to the genus *Bifidobacterium*, and one, B. longum, could be assigned at the species level. In the lactose vessel, five RSVs assigned to *Bifidobacterium* increased (at 24 h only) compared to that in the water vessel. Three of these also increased in the Lactobacillus LB vessel.

**TABLE 1:**
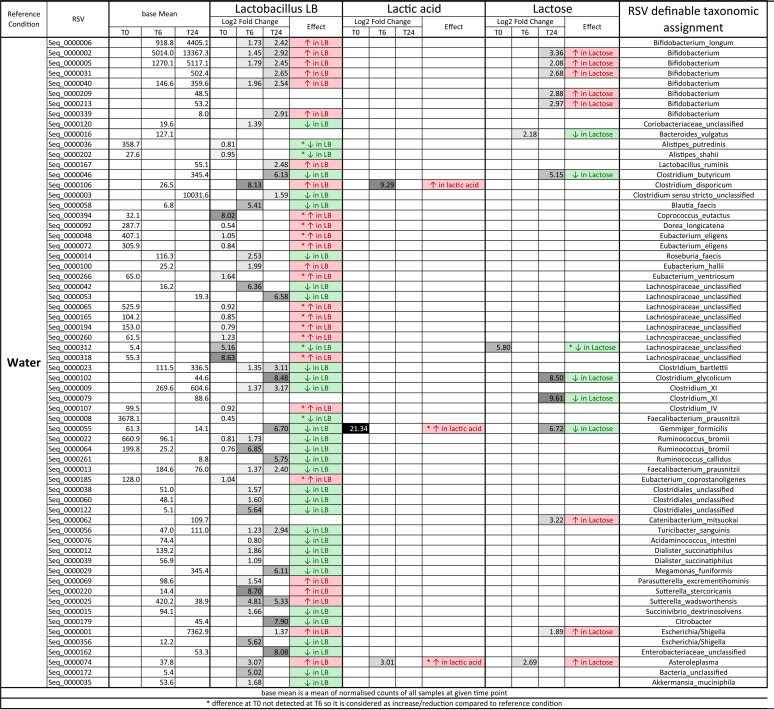
Abundance changes at the RSV level at the start of the experiment and after 6 and 24 h of fecal fermentation^*a*^

a Green indicates a reduction in abundance and red indicates an increase in abundance compared to reference condition (water). *, difference at 0 h that was not detected at 6 h and, therefore, is considered increase/reduction compared to the reference condition. Greyscale shading indicates the extent of log_2_ fold change, with white indicating no change and black indicating maximum log_2_ fold change. Base mean indicates mean normalized counts of all samples. Statistical significance for adjusted *P* values was set at *P* < 0.05; base mean and log_2_ fold change values are presented only for statistically significant changes due to treatment and/or time (empty cells show nonsignificant comparisons). Complementary comparisons between vessels are presented in Table S6.

Regrettably, replicate numbers (*n* = 3 to 4) limited the sensitivity with which we could detect statistically significant differences in α- and β-diversity (Tables S2 to S4). Nonetheless, α-diversity appeared (albeit insignificantly for most of the conditions) to decrease over time, as measured by Chao1 (Fig. 1A, Table S3) and Shannon indexes (Table S3). This decreasing trend in α-diversity most likely reflects the natural loss or reduction in levels of species that occurs in closed systems. Similarly, we did not observe statistically significant changes in α-diversity between the conditions tested at 0, 6, and 24 h (Table S2).

Microbiota diversity between individual samples (β diversity) was examined using a principal coordinate analysis (PCoA) approach (and was affected by the low replicate number). This PCoA plot revealed a clear visual shift (insignificant for most of the conditions) in microbiota composition during the 24 h of the experiment (Fig. 1B). Fermentation time had a major impact on the diversity, with PC1 explaining 66.07% of the variation, which is to be expected in a closed system. Initially, all vessels clustered together (Fig. 1B), but by the end of the fermentation, the microbiotas in individual vessels were distributed along PC2 (explaining 12.44% of variation). In particular, microbiota from the Lactobacillus LB vessel clustered at the top of the PC2 axis together with that from the lactose vessel. The microbiota from the lactic acid vessel clustered at the lower part of the PC2 axis together with that from the water vessel (Fig. 1B).

### Lactobacillus LB increases the absolute number of bifidobacteria in human fermented fecal communities.

**FIG 2:**
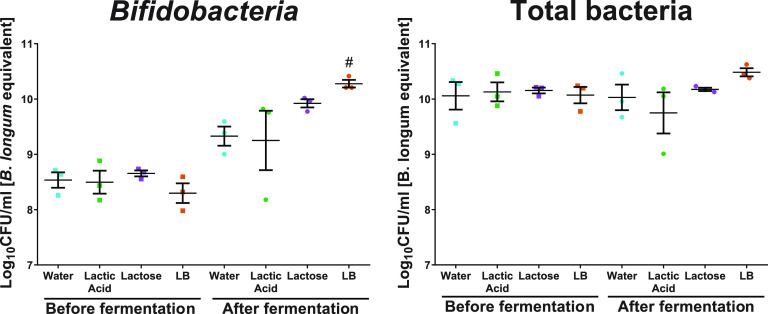
*Bifidobacterium* load and equivalent of total counts before (square) and after (circle) 24-h fecal fermentation in vessels supplemented with water (blue), lactic acid (green), lactose (purple), or Lactobacillus LB (LB; orange). Replicates 1, 2, and 4 are represented for a water vessel. The measurement of lower *Bifidobacterium* and total counts in lactic acid vessel corresponds to replicate A in Fig. 1. #, significant increase compared to the control vessel containing water supplementation at a given time point.

We used quantitative PCR (qPCR) to estimate the actual abundance of bifidobacteria in each vessel. At the start of the fermentation, there was no difference in *Bifidobacterium* levels (one-way analysis of variance [ANOVA], *F*_3,8_ = 0.912, *P* = 0.477) or in total bacterial load (one-way ANOVA, *F*_3,8_ = 0.074, *P* = 0.972) between any of the vessels (Fig. 2).

After 24 h of fermentation, there were differences in *Bifidobacterium* levels between vessels (Kruskal-Wallis test, *P* = 0.031) (Fig. 2). *Bifidobacterium* levels in water- and Lactobacillus LB-supplemented vessels differed significantly (manual *post hoc*, *P* value controlled for multiple comparisons, *P* = 0.007 < α/*k*), while *Bifidobacterium* levels in lactic acid and lactose showed intermediate levels and did not significantly differ from those of other vessels. At the same time, the 1.98-log and 1.27-log increases in *Bifidobacterium* within the Lactobacillus LB and lactose vessels, respectively, were a significant change (one-sample *t* test compared to 0; LB, *t*_2_ = 15.605, *P* = 0.004; lactose, *t*_2_ = 10.922, *P* = 0.008). In the remaining vessels (water and lactic acid), this change over time was not significant (one-sample *t* test compared to 0; water, *t*_2_ = 3.117, *P* = 0.089; lactic acid, *t*_2_ = 1.878, *P* = 0.201).

The different interventions had no significant effect on the total bacterial load following a 24-h fermentation (Welch test, *P* = 0.108; Brown-Forsythe, *P* = 0.285) (Fig. 2). There was also no change in total bacterial load over time (related-samples Wilcoxon signed rank test, *P* ≥ 0.109).

### Metabolite changes in human fermented fecal communities in the presence of a postbiotic, Lactobacillus LB.

An approximately 4-fold larger amount of NaOH was required, over approximately twice the time period, to control the pH in the presence of Lactobacillus LB compared to the lactic acid or water controls. This suggests that in the presence of a postbiotic, Lactobacillus LB, the fermentation process resulted in the production of larger amounts of acid and that acid production was maintained longer. To elucidate the nature of compounds present in the vessels before and after fermentation, metabolomics analysis was performed.

**FIG 3:**
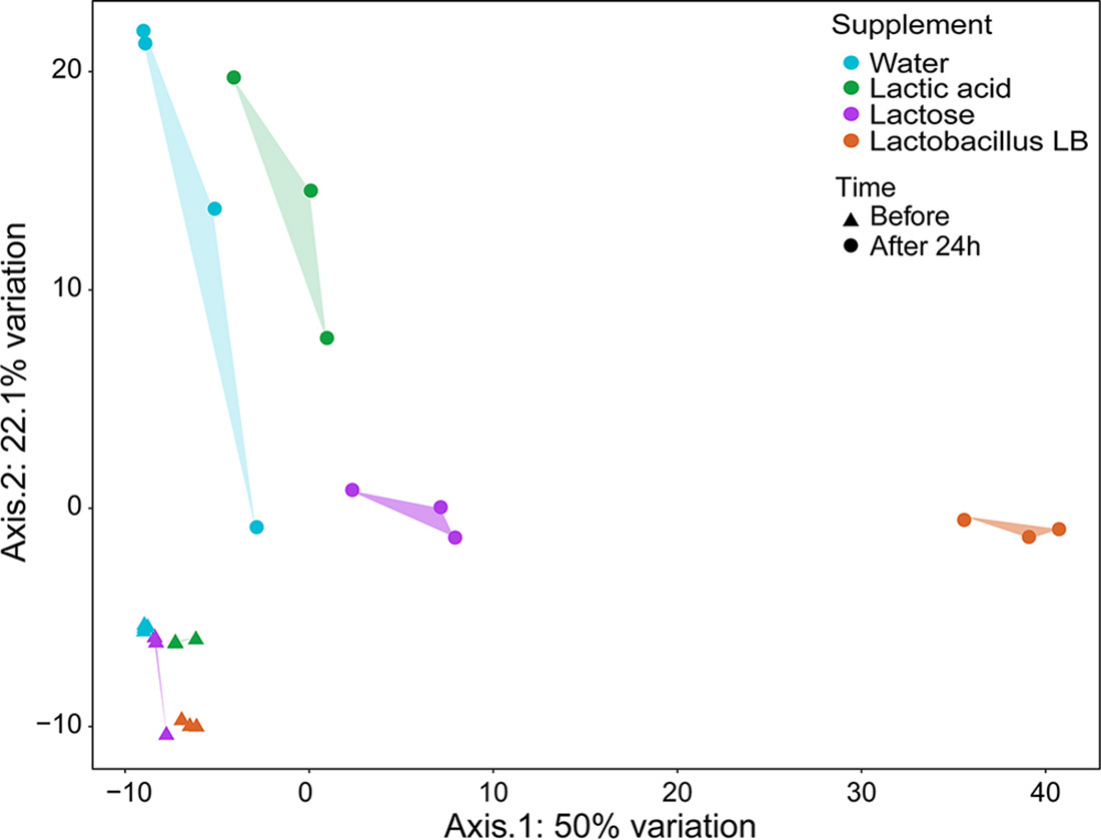
PCoA plot representing metabolic profiles based on the Euclidian distances of samples at the start (triangle) and after (circle) 24-h fecal fermentation in vessels supplemented with water (blue), lactic acid (green), lactose (purple), or Lactobacillus LB (orange). The graph was prepared based on the nonnormalized relative concentrations of the variables in reduced data sets for SCFA (6 compounds) and other metabolites (178 compounds).

Samples collected at the start of the fermentation from vessels supplemented with water, lactic acid, and lactose clustered tightly together, while samples from Lactobacillus LB vessels clustered separately (Fig. 3, Fig. S1). At the start of the fermentation, 33 annotated compounds had elevated levels in the Lactobacillus LB vessel compared to the water vessel (Fig. S2). Following 24 h of fermentation, the metabolite composition in all vessels had changed, with changes in Lactobacillus LB vessels being most pronounced (Fig. 3). At this stage, 39 annotated compounds had significantly higher levels in the Lactobacillus LB vessels than the water vessel (Fig. S3).

**(i) SCFAs.** The increase in acetic acid levels in Lactobacillus LB vessels was greater than that in water vessels (Fig. S4A) (Kruskal-Wallis test, *P* = 0.018; adjusted pairwise comparison, *P* = 0.026), while the increase in formic acid levels in Lactobacillus LB vessels was greater than in lactose vessels (ANOVA, *F*_3,12_ = 12.859, *P* = 0.001; Bonferroni *post hoc*, *P* = 0.003). Changes in propionic acid, butyric acid, isobutyric acid, and valeric acid did not differ between the vessels (*P* > 0.05).

**(ii) TCA cycle compounds.** During fermentation, the increase in levels of succinic acid (ANOVA, *F*_3,12_ = 16.695, *P* = 0.001; Bonferroni *post hoc*, *P* ≤ 0.036) and lactic acid (ANOVA, *F*_3,12_ = 90.337, *P* < 0.0005; Bonferroni *post hoc*, *P* ≤ 0.005) was greater in Lactobacillus LB vessels than all other vessels (Fig. S4B), while there was no difference between the vessels in the degree of change for the remaining tricarboxylic acid (TCA) cycle compounds.

**(iii) Amino acids and other metabolites.** With the exception of proline, the levels of all amino acids declined during the fermentation (Fig. S4C). Compared to water vessels, this reduction was more pronounced in Lactobacillus LB-supplemented vessels for leucine, isoleucine, threonine, tyrosine (ANOVA, *F*_3,12_ ≥ 6.078, *P* ≤ 0.015; Bonferroni *post hoc*, *P* ≤ 0.001), and tryptophan (Kruskal-Wallis test, *P* = 0.034; adjusted pairwise comparison, *P* = 0.020).

The levels of the majority of the remaining identified or annotated compounds showed a tendency to increase, while very few showed reductions in levels (Fig. S4D and E). Levels of benzoic acid, 2-hydroxybutyric acid, skatole, cinnamic acid, and 6 other compounds showed an increase in Lactobacillus LB vessels compared to water vessels. Levels of 3 compounds were reduced in the Lactobacillus LB vessels compared to water vessels.

### Effect of Lactobacillus LB and Lactobacillus LB fractions on the growth of bifidobacteria.

**FIG 4:**
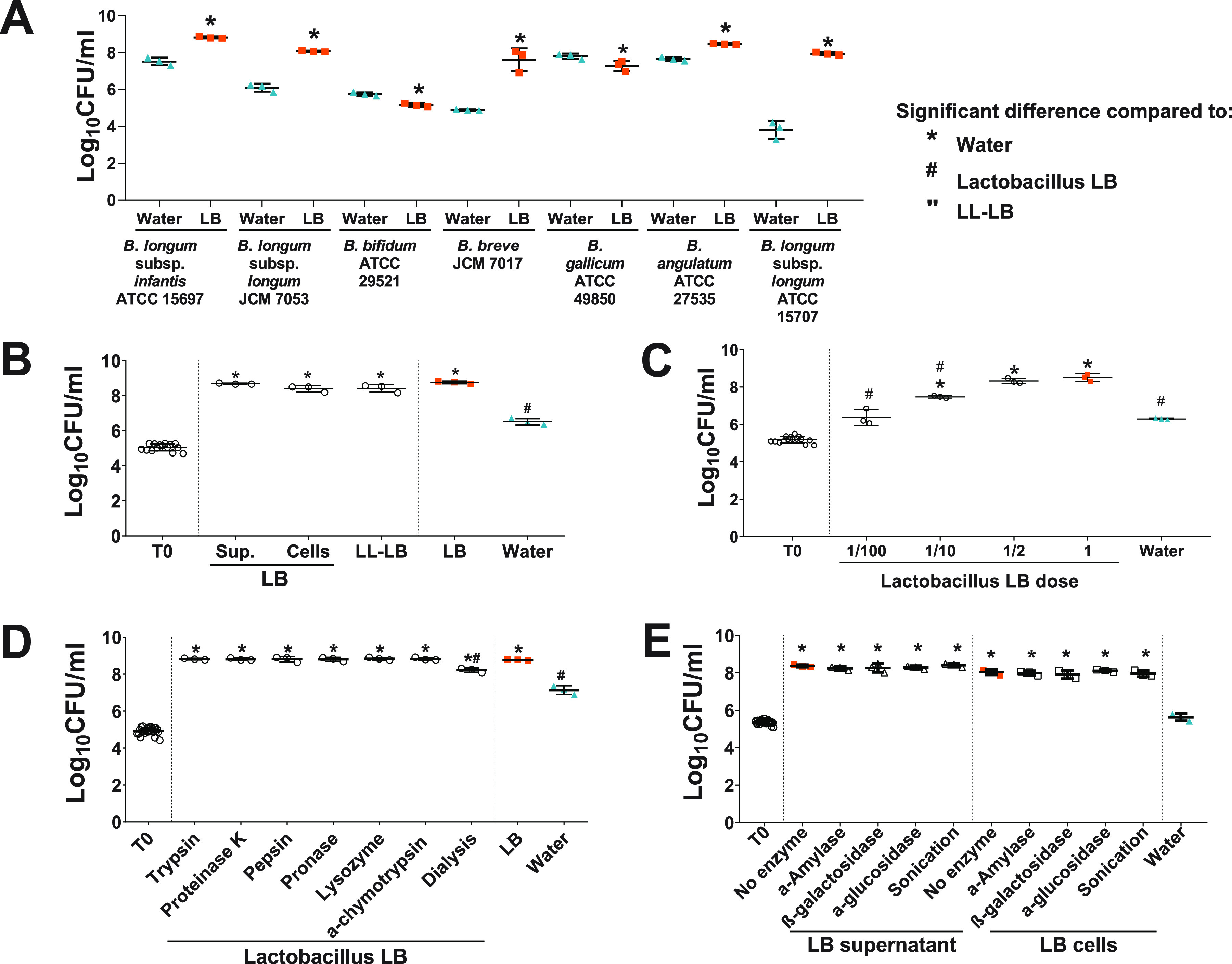
Twenty-four hours of growth of bifidobacterial in pure culture in 10× diluted media. (A) Effect of Lactobacillus LB (LB) on the growth of a range of infant- and adult-associated *Bifidobacterium* strains. Lactobacillus LB stimulated growth of Bifidobacterium longum subsp. *infantis* ATCC 15697 (B1; *t*_4_ = 18.555, *P* < 0.0005), Bifidobacterium longum subsp. *longum* JCM 7053 (*t*_4_ = 15.626, *P* = 0.003), Bifidobacterium breve JCM 7017 (*t*_4_ = 7.667, *P* = 0.016), Bifidobacterium angulatum ATCC 27535 (APC 329; *t*_4_ = 12.065, *P* < 0.0005), and Bifidobacterium longum subsp. *longum* ATCC 15707 (APC 2744; *t*_4_ = 14.603, *P* < 0.0005). The growth of only two strains, Bifidobacterium bifidum LMG 11041 (ATCC 29521; *t*_4_ = −7.432, *P* = 0.002) and Bifidobacterium gallicum ATCC 49850 (APC 838; *t*_4_ = −2.828, *P* = 0.047), was not stimulated. (B) Effect of Lactobacillus LB components, i.e., supernatant, cells, and low-lactose Lactobacillus LB (LL-LB) on B. longum subsp. *infantis* ATCC 15697 growth. (C) Effect of Lactobacillus LB dose on B. longum subsp. *infantis* ATCC 15697 growth. (D and E) Effect of enzymatically or physically treated Lactobacillus LB on B. longum subsp. *infantis* ATCC 15697 growth. *, significant change compared to water supplementation. #, significant change compared to Lactobacillus LB. “, significant change compared to LL-LB supplementation.

A postbiotic, Lactobacillus LB, stimulated the growth of a selection of both infant-associated and adult-associated bifidobacteria in pure culture (ANOVA, *F*_4,10_ = 108.650, *P* < 0.0005; Bonferroni *post hoc*, *P* < 0.0005) (Fig. 4A and B). Lactobacillus LB’s growth-stimulatory effect was studied in more detail using B. longum subsp. *infantis* ATCC 15697 as a model strain. Both soluble (supernatant) and insoluble (predominantly cells and cell debris) fractions of Lactobacillus LB stimulated the growth of the model strain to the same extent as Lactobacillus LB (Bonferroni *post hoc*, compared to water, all *P* values of <0.0005; compared to Lactobacillus LB, *P* = 1 and *P* = 0.165 [soluble and insoluble fractions, respectively]) (Fig. 4B). A low-lactose version of Lactobacillus LB (LL-LB) also showed growth stimulation comparable to that of Lactobacillus LB (Bonferroni *post hoc*, compared to water, *P* < 0.0005; compared to Lactobacillus LB, *P* = 0.225) (Fig. 4B).

Growth of *Bifidobacterium* depended on the Lactobacillus LB dose (ANOVA, *F*_5,11_ = 59.654, *P* < 0.0005) (Fig. 4C). Half-strength Lactobacillus LB stimulated growth to the same extent as Lactobacillus LB (Bonferroni *post hoc*, *P* = 1), 10× diluted Lactobacillus LB stimulated bifidobacterial growth to a lesser extent, and *Bifidobacterium* counts in 100× diluted Lactobacillus LB were the same as those in the water control (Bonferroni *post hoc*, *P* = 1).

The stimulatory activity was not affected by enzymatic treatments and a subsequent heat treatment used to inactivate enzymes (1 h at 92°C) (Fig. 4D and E). Lactobacillus LB preparations treated with a range of proteolytic enzymes as well as mock-treated Lactobacillus LB stimulated the growth of the bifidobacterial model strain (Fig. 4D) (ANOVA, *F*_8,18_ = 73.582, *P* < 0.0005; Bonferroni *post hoc* compared to water, all *P* values of <0.0005; Bonferroni *post hoc* compared to Lactobacillus LB, all *P* values of 1). Treatment with carbohydrate-digesting enzymes as well as mock treatment of supernatants and cell fractions of Lactobacillus LB preparations did not affect growth stimulation of the bifidobacterial model strain (Fig. 4E) (ANOVA, *F*_10,22_ = 85.783, *P* < 0.0005; Bonferroni *post hoc* compared to water, all *P* values of <0.0005; Bonferroni *post hoc* compared to supernatant or compared to the cell fraction of Lactobacillus LB, all *P* values of >0.05).

Lactobacillus LB that had been dialyzed to eliminate compounds under 1 kDa also stimulated the growth of the bifidobacterial model strain (Fig. 4D) (ANOVA, *F*_8,18_ = 73.582, *P* < 0.0005; Bonferroni *post hoc* compared to water, *P* < 0.0005). Although this stimulation was attenuated compared to that in Lactobacillus LB (ANOVA, *F*_8,18_ = 73.582, *P* < 0.0005; Bonferroni *post hoc* compared to Lactobacillus LB, *P* < 0.0005), there remained a significant stimulation compared to the water control. Sonication did not affect the activity of either supernatant or cell fraction of Lactobacillus LB (Fig. 4E) (ANOVA, *F*_10,22_ = 85.783, *P* < 0.0005; Bonferroni *post hoc* compared to water, all *P* < 0.0005; Bonferroni *post hoc* compared to supernatant or cell fraction of Lactobacillus LB, all *P* = 0.05).

**FIG 5:**
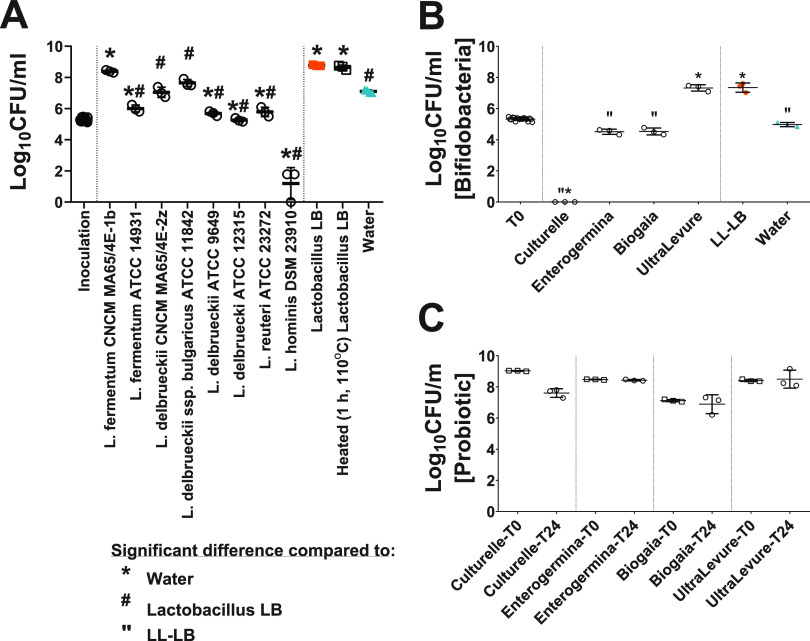
Twenty-four hours of growth of B. longum subsp. *infantis* ATCC 15697 in pure culture in 10× (A) or 15× (B and C) diluted media. (A) Effect of Lactobacillus LB-like preparations on growth. (B) B. longum subsp. *infantis* ATCC 15697 counts following commercial probiotic supplementation in 15× diluted media. No *Bifidobacterium* could be recovered from tubes supplemented with Culturelle. (C) Effect of test conditions on CFU counts of commercial probiotic. *, significant change compared to water supplementation. #, significant change compared to Lactobacillus LB. “, significant change compared to low-lactose Lactobacillus LB (LL-LB) supplementation.

There was a difference in 24-h growth levels of the bifidobacterial model strain in response to Lactobacillus LB-like preparations (Fig. 5A) (ANOVA, *F*_10,21_ = 141.368, *P* < 0.0005). Lactobacillus LB and *L*. *fermentum* CNCM MA65/4E-1b (one of the strains used to produce Lactobacillus LB) comparably stimulated *Bifidobacterium* growth (Bonferroni *post hoc*, compared to each other, *P* = 1; compared to water, *P* < 0.0005), while Limosilactobacillus fermentum ATCC 14931, Lactobacillus delbrueckii ATCC 9649, Lactobacillus delbrueckii ATCC 12315, and Limosilactobacillus reuteri ATCC 23272 (previously known as Lactobacillus reuteri ATCC 23272) did not promote growth (Bonferroni *post hoc*, all *P* values of <0.0005). Lactobacillus hominis DSM23910 supplementation had a clear killing effect on *Bifidobacterium*, as 6 or fewer CFU were recovered on plates. *L*. *delbrueckii* CNCM MA65/4E-2z (the second strain used to produce Lactobacillus LB) and Lactobacillus delbrueckii subsp. *bulgaricus* ATCC 11842 had no stimulatory activity (Bonferroni *post hoc*, *P* = 1 and *P* = 0.060, respectively). Additionally, the heat treatment used to inactivate Lactobacillus LB-like preparations (1 h at 110°C) had no impact on activity (Bonferroni *post hoc*, *P* = 1). Moreover, as in the case of LB preparation, 1-kDa dialysis of *L*. *fermentum* CNCM MA65/4E-1b (one of the producer strains) reduced the activity (data not shown).

### Effect of commercial probiotic preparations on bifidobacterial growth in a simple model.

After 24 h of incubation of bifidobacteria with different commercial probiotic products, bifidobacterial counts differed significantly between test conditions (Fig. 5B) (ANOVA, *F*_5,12_ = 574.535, *P* < 0.0005). Supplementation with Culturelle prevented the recovery of bifidobacteria (Bonferroni *post hoc* compared to any other treatment, *P* < 0.0005). Both Enterogermina and BioGaia had no impact on bifidobacterial counts, with counts comparable to those with water (Bonferroni *post hoc* compared to water, *P* = 0.192 and *P* = 0.242, respectively). Finally, Ultra Levure stimulated bifidobacterial growth to a level comparable to that of LL-LB (Bonferroni *post hoc* compared to water, *P* < 0.0005; Bonferroni *post hoc* compared to LL-LB, *P* = 1).

Over the 24 h of incubation, there were no changes in the numbers of the microorganisms originating from the commercial products (Fig. 5C): *Lacticaseibacillus rhamnosus* GG (previously Lactobacillus rhamnosus GG; Culturelle; Wilcoxon test, *P* = 0.109), Bacillus clausii (Enterogermina; *t*_2_ = 1.941, *P* = 0.192), Limosilactobacillus reuteri DSM 17938 (previously Lactobacillus reuteri DSM 17938; BioGaia; *t*_2_ = 0.595, *P* = 0.612), and *Saccharomyces boulardii* CNCM *I-745* (Ultra Levure; *t*_2_ = −0.253, *P* = 0.824).

## DISCUSSION

This study investigated the impact of a postbiotic, Lactobacillus LB, on the microbial composition of anaerobic batch cultures inoculated with human fecal samples. The most significant nonbacterial components of Lactobacillus LB are lactic acid, which is generated during initial fermentation by *L. fermentum* CNCM MA65/4E-1b and *L. delbrueckii* CNCM MA65/4E-2z, and lactose, which is added postproduction to facilitate the drying process. Consequently, as additional controls besides water, we included supplementation with lactic acid and lactose individually. We also developed a pure-culture model to study the impact of Lactobacillus LB and its related preparations on the anaerobic growth of *Bifidobacterium*.

Lactobacillus LB has bifidogenic activity that is perhaps best illustrated by the increase of the absolute abundance of *Bifidobacterium* in the *ex vivo* human fecal fermentation system. In previous studies, we demonstrated that Lactobacillus LB had an impact on murine microbiota (26, 27); however, as bifidobacteria were detected in only some of our animals, we saw no significant expansion of overall bifidobacterial populations. In other *in vivo* studies using mouse models, an expansion of the bifidobacterial population was reported following consumption of viable preparations of *Lactobacillaceae*. Specifically, the administration of fermented milk containing *Lacticaseibacillus casei* DN-114001 (previously Lactobacillus casei DN-114001) to BALB/c nursing mice and their offspring led to the culture-dependent expansion of bifidobacterial populations in the offspring. To maintain this effect, a continued supplementation postweaning was required (38). In another study, culture-dependent measures revealed that the bifidobacterial populations in both obese and nonobese BALB/c mice expanded after dosing with *Lacticaseibacillus casei* CRL 431 (previously Lactobacillus casei CRL 431) cells or milk fermented with *L*. *casei* CRL 431 (39).

As expected from a closed fermentation system, with no exchange of nutrients and waste, α-diversity was reduced in all the vessels over time. Initially, relatively low levels of *Escherichia*/*Shigella* (below 0.1%) increased in 6 h to 30 to 60% (particularly in lactic acid and Lactobacillus LB vessels), followed by a reduction to approximately 12% at 24 h. Lactic acid present in lactic acid and Lactobacillus LB vessels (directly supplemented as lactic acid or indirectly as a component of Lactobacillus LB) may have given advantage to lactate-utilizing (40) *Escherichia*. However, this was not the case for other lactate-utilizing bacteria, such as *Veillonella* (41, 42), which maintained rather stable relative abundance under all conditions tested.

In turn, lactose present in lactose and Lactobacillus LB vessels (directly supplemented as lactose or indirectly as a component of Lactobacillus LB) may have favored lactose-utilizing bacteria, including bifidobacteria, lactic acid bacteria (LAB), and members of *Enterobacteriaceae* (43–45). While the effect of lactose on bifidobacterial relative abundance is similar to that of Lactobacillus LB (an increase from 5% to 66% in lactose and to 75% in Lactobacillus LB), the absolute increase in bifidobacteria was seen only in Lactobacillus LB. Furthermore, low-lactose preparations (LL-LB and single-strain preparation) also promoted the growth of bifidobacteria (see below), indicating a lactose-independent growth stimulation.

At the end of fermentation in lactose-supplemented vessels, besides bifidobacteria, we also observed an increase in relative abundance of *Escherichia*/*Shigella* (46 and described above), *Megamonas* (46) (from 0.3% to 1.1%, compared to 2.8% in water and a drop to 0.03% in Lactobacillus LB), and *Streptococcus* (47) (from 0.4% to 2.8%, compared to 2.3% in water and a stable level of 0.6% in Lactobacillus LB). Contrary to expectations, there was no increase in relative abundance of known lactose fermenters, *Lactobacillus* and *Lactococcus* (47), in lactose vessels (as well as in other vessels). Before fermentation, the relative abundance of *Enterobacter*, *Enterococcus*, *Klebsiella*, and *Citrobacter* was extremely low, possibly explaining the lack of their expansion in the lactose vessels (and/or remaining conditions).

Another important intrinsic factor to consider is the presence of starch in all vessels. Starch-based carbohydrates were recently shown to have bifidogenic and butyrogenic properties (48). This may correspond to the modest increase (albeit nonsignificant and limited compared to Lactobacillus LB) in relative and absolute bifidobacteria in the water vessels. Moreover, at the beginning of fermentation, samples had a high portion of butyrate-producing species (33), particularly *Faecalibacterium* (17.1%) and *Roseburia* (11.7%). However, during the fermentation, both *Faecalibacterium* and *Roseburia* levels rapidly decreased, falling below 0.5% after 6 h. This is consistent with the low levels of butyrate detected in vessels both before (since carryover of bacterial metabolites was limited by washing of fecal inoculum) and after the fermentation.

We observed increased levels of acetic acid, formic acid, and lactic acid in the vessels at the end of the fermentation. This is consistent with the expansion of bifidobacteria in the Lactobacillus LB vessels, as these acids are end products of bifidobacterial metabolism (49, 50). The presence of either bifidobacteria or SCFA generally has been considered beneficial to host health (33). In addition, increased acid production could lead to a degree of acidification of the environment. The pH naturally changes along the gastrointestinal (GI) tract and has been reported to affect microbial and metabolic interactions (51). It is possible that a decrease in pH following Lactobacillus LB supplementation has beneficial effects on the host. Lower pH may prioritize the growth of acid-tolerant/acid-producing species, such as LAB and some *Bifidobacterium* spp., which are mostly characterized by a weak acid tolerance (except for *B*. *lactis* and *B*. *animalis* strains [52]). These beneficial effects may include a reduction in diarrhea, promotion of cholesterol absorption, and/or immunomodulation (14). Ilhan et al. (51) reported that microbiota structure has a greater dependence on pH than carbon source (glucose, fructose, and cellobiose).

Prior to fermentation, both the microbiome and the metabolite analyses were tightly clustered, confirming the reproducibility of the preparations. As expected, we saw no differences between vessels in terms of microbiome composition. However, the Lactobacillus LB vessels showed altered metabolite profiles at the start of the fermentation, predominantly in terms of elevated levels of amino acids, which set this condition apart from the controls and reflects the complex mixture of components in the Lactobacillus LB preparation.

Overall, our results from a *Bifidobacterium* single-strain model confirm that Lactobacillus LB stimulates the growth of bifidobacteria (and possibly other gut bacteria to a lesser extent). This is perhaps best exemplified by growth stimulation of five of seven tested *Bifidobacterium* strains isolated from both infants and adults. A lack of growth stimulation was observed for *B*. *bifidum* LMG 11041 and *B*. *gallicum* ATCC 49850. Both strains showed poor overnight growth and required the use of a higher inoculum for the bifidogenic assay. Nonetheless, in the bifidogenic assay, they responded oppositely; LMG 11041 was inactivated under the test conditions (2-log inactivation in the water control) while ATCC 49850 grew well, also showing growth in the water control (7.8 log after 24 h). Strain-specific responses of bifidobacteria have been reported previously, particularly in their abilities to utilize prebiotics (53–56).

Dead cells, along with viable cells, are very likely to be found in probiotic products, since probiotic die-off is unavoidable, especially in products with a long shelf-life (23). Recent considerations on the role of dead cells in the beneficial effects of probiotics (23), together with the wider commercial application of probiotics, encouraged us to use our experimental system to compare the bifidogenic effect of a postbiotic, Lactobacillus LB, with commercial probiotics. Three commercially available bacterial products did not stimulate *Bifidobacterium* growth. These included spores of *B*. *clausii*, cells of L. reuteri DSM 17938, and L. rhamnosus GG. Interestingly, the L. rhamnosus GG preparation contains inulin, yet despite the presence of this well-known prebiotic, we observed the inactivation of *Bifidobacterium*. In line with those observations, none of the five preparations generated by the non-Lactobacillus LB *Lactobacillaceae* were able to stimulate bifidobacterial growth to the same extent as Lactobacillus LB. Further investigation demonstrated that of the two strains used in Lactobacillus LB production, only the *L*. *fermentum* CNCM MA65/4E-1b preparation stimulates *Bifidobacterium* growth to an extent that is comparable to that of Lactobacillus LB.

A yeast-based preparation containing *S. boulardii* CNCM *I-745* did stimulate *Bifidobacterium* growth to an extent similar to that of Lactobacillus LB. This was not unexpected, as it has been previously shown that a 0.1% supplementation with cell wall extracts from baker’s yeast (Saccharomyces cerevisiae), containing 99.54% β-glucans, stimulated bifidobacterial growth in infant formula (57).

To investigate the uniqueness and specificity of the observed activity, a series of physical and enzymatic tests were performed. Lactobacillus LB activity was found to be dose dependent, heat stable, and not affected by either proteolytic or carbohydrate digesting enzymes. However, the attenuated activity of 1-kDa dialysis of Lactobacillus LB suggests that some of the activity is associated with small compounds (possibly bacterial metabolites, signaling molecules, and/or soluble cell membrane fractions), particularly as both Lactobacillus LB supernatant and cell fractions showed comparable stimulation of bifidobacterial growth.

*In vitro* distal colon models can provide important insights into the responses of gut microbiota to preparations tested using them. As with all *in vitro* models, there are limitations in translating findings to the *in vivo* situation, in particular the effects of the digestive tract on the test article, which includes host responses. In addition, the statistical power of microbiota composition analysis was also limited by a limitation in the number of replicates, which restricted the range of microbiota changes that could be analyzed. We have provided evidence for the presence of a bifidogenic factor in Lactobacillus LB, although we do recognize that a clinical study would be required to ensure the translation of our findings.

This study provides evidence in support of the potential effects of Lactobacillus LB, a postbiotic generated by heat treatment of *L. fermentum* CNCM MA65/4E-1b and *L. delbrueckii* subsp. *delbrueckii* CNCM MA65/4E-2z fermentate that includes both biomass and metabolites, on the human colonic microbiota, specifically on the microbiota metabolites and the growth of bifidobacteria. This was found to be an effect that was not generalized to probiotic bacteria in our investigation. Preparations containing heat-treated microorganisms, postbiotics, or pharmabiotics appear to be effective interventions in impacting the microbiome to positively affect host health.

## MATERIALS AND METHODS

### Lactobacillus LB preparations.

Lactobacillus LB powder (batch 16L202, prepared 18 February 2016; Lyophilisate Actif LB), provided by Adare Pharmaceuticals, was used for feeding fecal fermentation vessels. In growth experiments, reconstituted Lactobacillus LB powder (0.34 g/ml) or LL-LB solution was used. Lactobacillus LB contains lactose, which is added postproduction to facilitate the drying process, while LL-LB is in liquid form and has no added lactose. Each preparation contained 5 × 10^9^ to 1 × 10^10^ cell bodies per ml of the solution unless stated otherwise.

In previous *in vivo* studies (26, 27), rodent diet containing the same API was termed ADR-159. For paper readability, all preparations containing this API are referred to as Lactobacillus LB.

### Measurement of lactose and lactic acid concentration in LB preparation.

Concentrations of lactose, glucose, succinate, lactate, formate, acetate, and propionate in LB preparation and producer strains supernatant were measured using high-performance liquid chromatography (HPLC).

### Fecal fermentation.

**(i) Preparation of FSI.** The FSI was prepared based on the modification of a recently developed method (1). Briefly, consented volunteers/donors (*n* = 5) adhered to strict criteria; all donors were healthy adults and did not take antibiotics for 6 months before donation. This study was approved by the Clinical Research Ethics Committee (APC1002). The fecal samples from donors were collected into plastic containers, placed in zip bags with generators under anaerobic conditions (GENbox anaer; bioMérieux, France), and stored at 4°C. Within, on average, 9 h, fecal samples were transferred into an anaerobic chamber (Don Whitley, West Yorkshire, UK) under an anoxic atmosphere (10% H_2_, 0% O_2_, 0% N_2_). The feces were pooled into a large stomacher bag with a 70-μm filter insert (Sparks Lab Supplies, Ireland) in an anaerobic chamber. Four hundred milliliters of 50 mM phosphate buffer with 0.05% (wt/vol) l-cysteine hydrochloride (Sigma-Aldrich, Ireland), pH 6.8 (further referred to as phosphate buffer), was added to the stomacher bag, followed by manual sample homogenization. The filtered slurry was then centrifuged at 4,000 × *g* for 25 min in a Sorvall SLA-3000 centrifuge and resuspended in 400 ml phosphate buffer, again in an anaerobic cabinet. Next, the resulting solution underwent a second centrifugation (4,000 × *g* for 25 min), followed by resuspension in 400 ml phosphate buffer. The resulting fecal bacterial suspension was supplemented with 200 ml of glycerol, aliquoted, and frozen at −80°C for 1 to 9 weeks until use (here referred to as FSI). Except for the centrifugation step, all processing took place in the anaerobic cabinet. Before the use of FSI, the aliquots were thawed at 37°C over 0.5 to 1 h before inoculation into fermentation vessels.

**(ii) Distal colon model: fecal fermentation.** Starch-supplemented fecal medium was prepared as previously described (58), with the final concentration in the fermentation vessel (total volume, 200 ml) per liter being 2 g peptone, 2 g yeast extract, 0.76 g NaCl, 0.04 g K_2_HPO_4_, 0.04 g KH_2_PO_4_, 0.007 g CaCl_2_·2H_2_O, 0.01 g MgSO_4_·7H_2_O, 2 g NaHCO_3_, 2 ml Tween 80, 0.5 g l-cysteine-HCl, 0.5 g bile salts, 10 g soluble starch, 0.05 g hemin (dissolved in three drops of 1 M NaOH), and 10 μl vitamin K_1_ (Sigma-Aldrich). A volume of 180 ml of this base medium was supplemented with either Lactobacillus LB at 3.4 g/100 ml (equivalent to 10 capsules/sachets of Lacteol [10bn; 340 mg] in 100 ml) or equivalent amounts of lactic acid (30 mM) or lactose (36 mM) or water as a control. The medium was added to fermentation vessels of the MultiFors system (Infors, UK), its pH was adjusted to 6.8, and each vessel was sparged with oxygen-free N_2_ for at least 120 min to ensure anaerobic conditions were established. A volume of 12.5 ml FSI was used to inoculate each vessel, bringing the final volume in each vessel to 200 ml. Fermentations were performed over 24 h at 37°C, maintained at a constant pH of 6.8 by the automatic addition of 1 M NaOH or 1 M HCl, sparged with oxygen-free N_2_, and continuously stirred at 200 rpm. Samples were withdrawn from each of the vessels at 0 h, 1 h, 2 h, 3 h, 4 h, 5 h, 6 h, and 22 h and after 24 h of fermentation and stored at −80°C until processing. Each of the conditions was tested at least in triplicate.

### DNA isolation.

DNA isolation from fecal fermentation samples was performed using a QIAamp fast DNA stool minikit (Qiagen, Germany) according to the manufacturer's recommendations, with minor modifications, increasing the volume of used bead-beaten (FastPrep-24; MP Biomedicals, United States) solution to 600 μl and decreasing the final elution volume to 30 μl Tris-acetate-EDTA. The assessment of DNA quantity and quality was performed by measurement of DNA concentration using a Qubit dsDNA BR assay kit (Invitrogen) and running 5 μl sample on a gel for quality assessment.

### 16S Metagenomics: microbiota analysis.

**(i) DNA amplification, indexing, normalization, and sequencing.** Library preparation was performed as previously described (27). V3 and V4 region of 16S genes were amplified using Phusion polymerase master mix (Thermo Scientific) and V3-V4 (forward, 5′-TCGTCGGCAGCGTCAGATGTGTATAAGAGACAGCCTACGGGNGGCWGCAG-3′; reverse, 5′-GTCTCGTGGGCTCGGAGATGTGTATAAGAGACAGGACTACHVGGGTATCTAATCC-3′) primers (98°C for 30 s, 25 cycles of 98°C for 10 s, 55°C for 15 s, and 72°C for 20 s, and then 72°C for 5 min). Amplicons were checked for quality and quantity by a Qubit double-stranded DNA (dsDNA) HS assay kit (Invitrogen) and running on the gel and were cleaned using AMPure XP magnetic beads (Beckman Coulter). A total of 5 μl of the cleaned amplicon was used as a template for index PCR using Phusion polymerase master mix and the Nextera XT index kit (95°C for 30 s, 8 cycles of 95°C for 30 s, 55°C for 30 s, 72°C for 30 s, and 72°C for 5 min). Indexed amplicons were cleaned using AMPure XP magnetic beads and checked for quality and quantity by the Qubit dsDNA HS assay kit and running on the gel. All samples were normalized in water to 4 nM, followed by pooling 5 μl of each sample and sending to GTAC (Germany) for Illumina MiSeq sequencing.

**(ii) 16S data analysis.** The quality of the raw reads was visualized with FastQC v0.11.3. The reads were then imported into R v3.3.0 for analysis with the DADA2 package (v1.03) (59). Errors introduced during the sequencing process were corrected to generate ribosomal sequence variants (RSVs). These were exported and further chimera filtered using both the *de novo* and reference-based chimera filtering implemented in USEARCH v8.1.1861 with the ChimeraSlayer gold database v20110519. The remaining RSVs were classified with mothur v1.38 (60) against the RDP database version 11.4 and classified with SPINGO to species level (61). Only RSVs with a domain classification of *Bacteria* or *Archaea* were kept for further analysis. A phylogenetic tree of the RSV sequences rooted on the midpoint was generated with FastTree (62). Alpha diversity and beta diversity were generated using PhyloSeq v1.16.2, which also was used for a principle coordinate analysis as implemented in Ape v3.5. Differential abundance analysis was carried out with DESeq2 v1.12.4 (63) for RSV level and Wilcoxon tests for phylum to genus. *P* values were adjusted where necessary using the Benjamini-Hochberg method. All visualization in R was performed with ggplot2 v2.2.1.

A limited number of statistically significant differences were detected using Wilcoxon test, most likely due to the limited number of samples per group. Additional replicates (four in total) for the water vessel and, consequently, higher power for this condition may explain seldom-observed statistical differences for this condition.

### qPCR.

Isolated DNA was used to relatively quantify total microbial load and bifidobacterial load. Reactions were run on a 384-well LightCycler 480 PCR (Roche) using LightCycler 480 plates and adhesive cover (Roche). Each 15-μl reaction mixture contained 6.5 μl water, 7.5 μl 2× SensiFAST SYBR No-ROX master mix (Bioline), 0.3 μl of each of 10 μM primers (forward and reverse), and 1 μl of DNA sample. No template controls (NTC) were prepared using water instead of DNA. Samples were diluted 100 times before use. Each reaction was run in quadruplicates. Primers were used for total counts (U16SRT-F, 5′-ACTCCTACGGGAGGCAGCAGT-3′; U16SRT-R, 5-TATTACCGCGGCTGCTGGC-3′) (64) and for bifidobacterial quantification (Bif-xfp-F1, 5′-CGTCCGTTCTACCCGATG-3′; Bif-xfp-R1, 5′-GGTCTTCTTGCCGTCGAT-3′). Cycling parameters were 95°C for 5 min, followed by 45 cycles of 95°C for 10 s, 60°C for 30 s, and 72°C for 30 s. A melting curve analysis (60 to 97°C) was included at the end of every program to eliminate nonspecific amplification. Crossing point (Cp) values and melting temperature were calculated automatically using instrument software.

The efficiency of primers was checked using 10-fold dilutions of DNA isolated from 10 ml overnight grown culture of Bifidobacterium longum subsp. *infantis* ATCC 15697, resulting in an E value equal to 95.7% and 95.5% for U16SRT and Bif-xfp primers, respectively. The same dilution range was used as a standard curve for both microbial groups to correlate the number of CFU/ml with Cp values. At the same time, the number of 16S rRNA copies/ml was calculated based on 4 copies of 16S gene in the B. longum subsp. *infantis* ATCC 15697 genome (based on the *rrn*DB database, a mean copy number for a bacterial genome is 4.9 [https://rrndb.umms.med.umich.edu/; entry 12 December 2018]).

### Metabolite analysis.

Samples were defrosted on ice and centrifuged in a tabletop device for 1 min at maximal speed. The supernatant was filtered through a 0.2-μm filter, transferred into glass vessels, and stored at −20°C until measurement. Sample analysis was carried out by MS-Omics as follows.

For SCFA analysis, samples were acidified using hydrochloride acid, and deuterium-labeled internal standards were added. All samples were analyzed in a randomized order. The analysis was performed using a high-polarity column (GC cap column, 30 m by 0.25 mm by 0.25 μm; Zebron ZB-FFAP) installed in a gas chromatograph (GC; 7890B; Agilent) coupled with a quadrupole detector (5977B; Agilent).

For the analysis of GC metabolites, samples were derivatized with methyl chloroformate using a slightly modified version of the protocol described by Smart et al. (65). All samples were analyzed in a randomized order. The analysis was performed using the same GC device.

In both cases, the system was controlled by ChemStation (Agilent). Raw data were converted to netCDF format using ChemStation (Agilent) before the data were imported and processed in Matlab R2014b (Mathworks, Inc.) using the PARADISe software described by Johnsen et al. (66).

Obtained relative concentrations of the variables in reduced data sets for SCFA and other metabolites were directly used to calculate distance-based metrics (Euclidean, Minkowski, and Manhattan) and similarity-based metrics (Jaccard) that were recommended previously for metabolite analysis (67). Calculations and visualization were performed using R v3.6.2. with packages PhyloSeq v1.30.0 and ggplot2 v3.2.1.

### Pure culture, a simple model.

**(i) Preparation of *Bifidobacterium* inoculum.** A volume of 100 μl of −80°C stock of a *Bifidobacterium* strain (Table 2) was injected into anaerobic Hungate tubes containing 10 ml of MRS broth (Difco, BD) supplemented with l-cysteine (final concentration, 0.6 g/liter) and resazurin (final concentration, 1 mg/liter). Tubes were incubated overnight at 37°C. A volume of 100 μl of 100× diluted overnight culture was used as an inoculum unless stated otherwise. For *B. bifidum* LMG 11041 and *B. gallicum* ATCC 49850, 100 μl of 10× diluted overnight culture was used due to the poor overnight growth.

**TABLE 2:** Strains used in this study

Strain	Isolation	Use
*L. fermentum* CNCM MA65/4E-1b	Healthy human feces	Producer
*L. delbrueckii* subsp. *delbrueckii* CNCM MA65/4E-2z	Healthy human feces	Producer
L. reuteri ATCC 23272 (APC 2482, DSM 20016)	Intestine of adult	Comparison
*L. delbrueckii* subsp. *bulgaricus* ATCC 11842 (APC 2493, DSM 20081)	Bulgarian yoghurt	Comparison
*L. delbrueckii* ATCC 9649 (APC 2421, DSM 20074)	Sour grain mash	Comparison
*L. delbrueckii* ATCC 12315 (APC 2516, DSM 20072)	Emmental cheese	Comparison
*L. fermentum* ATCC 14931 (APC 249, DSM 20052)	Fermented beets	Comparison
*L. hominis* DSM23910 (APC 2512)	Human intestine	Comparison
B. longum subsp. *infantis* ATCC 15697	Intestine of infant	Main target
B. longum subsp. *longum* JCM 7053	Infant feces	Target
*B. bifidum* ATCC 29521 (LMG 11041)	Infant feces	Target
*B. breve* JCM7017	Human feces	Target
*B. gallicum* ATCC 49850 (APC 838)	Human feces	Target
*B. angulatum* ATCC 27535 (APC 329)	Human feces	Target
B. longum subsp. *longum* ATCC 15707 (APC 2744)	Intestine of adult	Target

**(ii) Bifidogenic assay.** A base medium was composed of 10× diluted MRS broth supplemented with l-cysteine (final concentration, 0.6 g/liter) and resazurin (final concentration, 1 mg/liter). A 10× dilution factor was selected, as it supported no or minimal growth of *Bifidobacterium* in the absence of growth-stimulating supplements (water control). The dilution factor was set for each of two batches of powdered media used in this paper; thus, in some of the experiments, a 15× diluted medium was used. A volume of 9 ml of base medium in an anaerobic Hungate tube was supplemented with 1 ml of test solution (one of the Lactobacillus LB preparation) or one of the controls (predominantly water). Next, the Hungate tube was inoculated, and the *t*_0_ sample was collected using a syringe and needle to limit oxygen access. The Hungate tube was incubated at 37°C for 24 h before sample collection. Collected samples were serially diluted in phosphate-buffered saline, and 100 μl was plated in duplicate on fresh MRS agar plates supplemented with l-cysteine (final concentration, 0.6 g/liter). Plates were incubated for at least 48 h at 37°C in jars with anaerobic generators (bioMérieux) before colony counting.

### Lactobacillus LB treatments.

**(i) Enzymatic treatments.** A volume of 5 ml of Lactobacillus LB solution was supplemented with 10 mg of proteinase K (with the addition of 1 mM CaCl_2_·2H_2_O), trypsin, pepsin, pronase, lysozyme, or α-chymotrypsin. A volume of 5 ml of either cell or supernatant fractions of Lactobacillus LB solution was supplemented with 1,000 U cellulase, 25 U α-glucosidase, 1,000 U α-amylase, and 125 U β-galactosidase. Tubes were incubated for 4 h at 37°C with shaking followed by 1 h at 92°C. Enzymatically treated Lactobacillus LB, cells, and supernatant fractions were stored at 4°C until tested in the bifidogenic assay. The pH of supernatants treated with α-amylase and β-galactosidase was increased to 7.16 before enzyme addition, adjusted back to the original pH, and filtered after incubation. A volume of 1 ml of enzymatically treated cells or supernatant was used in the bifidogenic assay (10× diluted media).

**(ii) Physical treatment: dialysis.** A total of 5.1 g of Lactobacillus LB powder resuspended in 15 ml water was transferred into a washed 1-kDa dialysis tube (Pur-A-Lyser Magna 1000; Sigma). The tube was then placed in 4.5 liters of demineralized water and incubated with steering at 4°C for 4 days. Water was changed daily. The content of the tube was transferred into a Hungate tube and stored at 4°C until use in the bifidogenic assay (10× diluted media).

**(iii) Physical treatment: sonication.** Lactobacillus LB solution was centrifuged (Servall ST 16R, with rotor TX-400; 5 min at 4,696 × *g*), the supernatant was filtered, and the cell pellet was washed twice. Supernatant and cell fractions were sonicated for 4 h (Ultrawave U300H, UK) and stored at 4°C until use in the bifidogenic assay (10× diluted media).

### Preparation of Lactobacillus LB-like preparations.

*Lactobacillaceae* strains (Table 2) were streaked from −80°C stocks onto MRS plates. A volume of 10 ml MRS broth was inoculated with a single colony and incubated anaerobically overnight at 37°C. A 1% inoculum was used for flasks with MRS broth supplemented with l-cysteine (final concentration, 0.6 g/liter). Media for growth of *L*. *delbrueckii* CNCM MA65/4E-2z were supplemented with 1% pepsin from casein to facilitate its growth requirements. Following anaerobic overnight incubation at 37°C, the content of flasks was distributed into large petri dishes and placed at −80°C until freeze-drying (−100°C at 0.06 mBar; Scanvac CoolSafe). Freeze-dried content was scraped off the plates and resuspended to 0.34 g/ml water before heat treatment for 1 h at 110°C (heat treatment applied during Lactobacillus LB preparation) and stored at 4°C until use.

### Effect of commercial products on bifidobacteria.

Solutions of commercial products were prepared in concentrations corresponding to their daily dose. In particular, the content of one capsule of Culturelle (10^10^ CFU; cells of *Lacticaseibacillus rhamnosus* GG [previously Lactobacillus rhamnosus GG] and inulin) was resuspended in 1 ml of water. The content of three flacons of Enterogermina (6 × 10^9^ CFU; spores of Bacillus clausii SIN, *B*. *clausii* O/C, *B*. *clausii* T, and *B*. *clausii* N/R; Sanofi) was centrifuged (Servall ST 16R, with rotor TX-400; 10 min at 4,696 × *g*) and resuspended in 1 ml of water. Five drops of BioGaia (10^8^ CFU of Lactobacillus reuteri DSM 17938) were resuspended in 1 ml of water. The content of one capsule of Ultra Levure (BIOCODEX; 200 mg of *Saccharomyces boulardii* CNCM *I-745*) was resuspended in 1 ml of water. A quantity of Lactobacillus LB containing 10^10^ bacterial cell bodies of *L*. *fermentum* CNCM MA65/4E-1b and *L*. *delbrueckii* CNCM MA65/4E-2z was resuspended in 1 ml of water (equivalent to 1 capsule of Lacteol 10bn).

A volume of 1 ml of commercial product solution was used to test its effect in the bifidogenic assay, with the following modifications. The modification to base media was the use of 15× diluted MRS rather than 10× diluted MRS (a new batch of powdered medium was used for this experiment). For modifications to plating conditions, serially diluted samples were plated on MRS plates with l-cysteine (0.6 g/liter), cycloheximide (70 mg/liter), and mupirocin (50 mg/liter). The selective enumeration of product counts was performed for L. rhamnosus GG (subtracting bifidobacterial counts from MRS counts), *B*. *clausii* (brain heart infusion, aerobic incubation), L. reuteri DSM 17938 (MRS plus tetracycline [30 μg/ml], anaerobic), and *Saccharomyces boulardii* CNCM *I-745* (Sabouraud [4% dextrose], aerobic incubation).

### Data availability.

Sequencing data discussed in this publication have been deposited in the Sequence Read Archive (SRA) and are accessible under accession number PRJNA545405.

## Supplementary Material

Supplemental file 1

Supplemental file 2
