# Supplementary material for: A Postbiotic Consisting of Heat-Treated Lactobacilli Has a Bifidogenic Effect in Pure Culture and in Human Fermented Fecal Communities
Source: Appl Environ Microbiol. 2021 Mar 26;87(8):e02459-20. doi: 10.1128/AEM.02459-20 (PMC8091120; doi:10.1128/AEM.02459-20)
Supplement: Supplemental file 2 [file AEM.02459-20-s0001.pdf]

SUPPLEMENTARY FIGURES

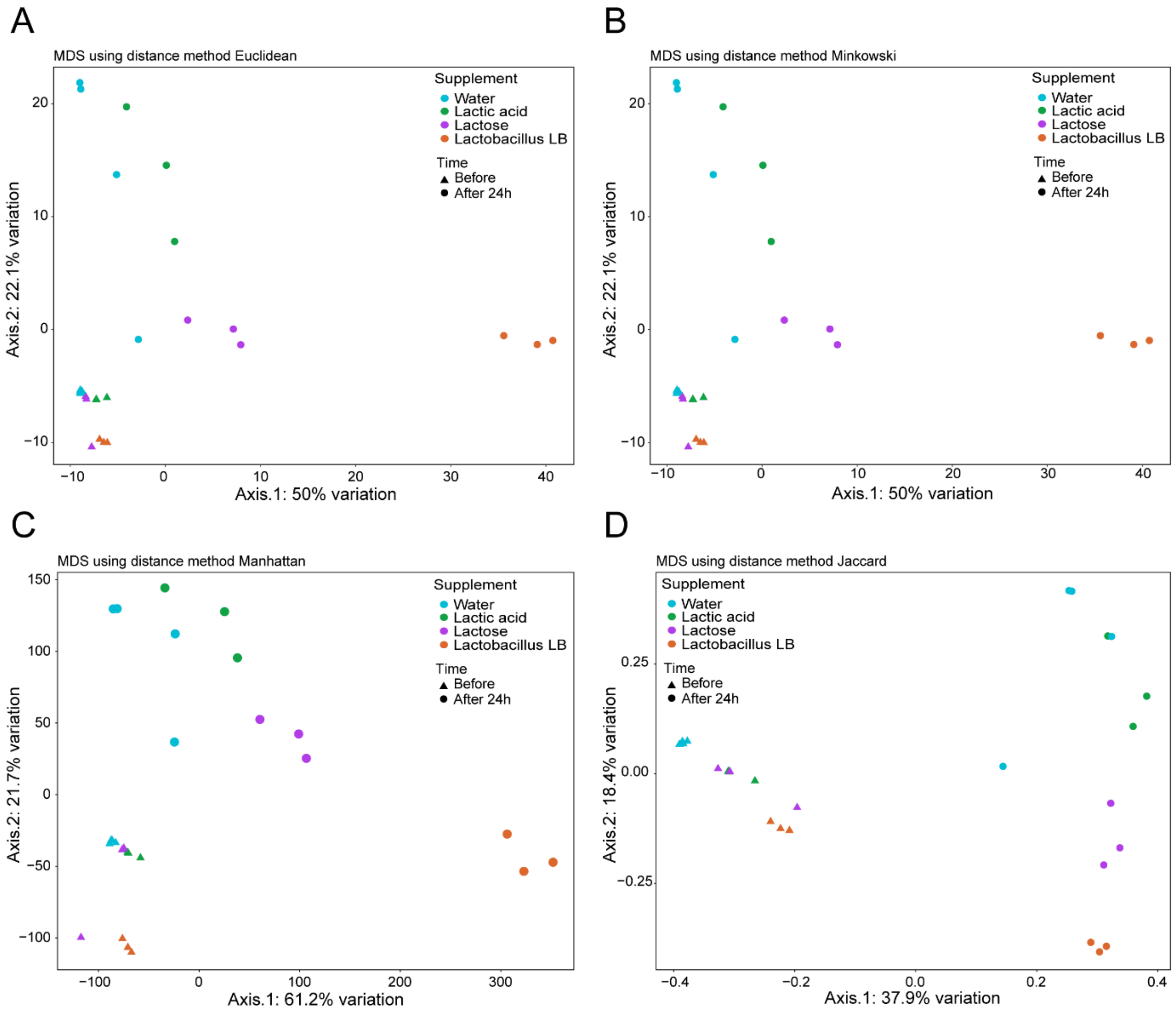

Figure S1. PCoA plot representing metabolic profiles based on the (A) Euclidian, (B) Minkowski, (C) Manhattan and (D) Jaccard distances of samples at the start (triangle) and after (circle) 24-hour faecal fermentation in vessels supplemented with water (blue), lactic acid (green), lactose (purple) or Lactobacillus LB (orange). Graph prepared based on the non-normalised relative concentrations of the variables in reduced datasets for SCFA and other metabolites (6 + 178 compounds).

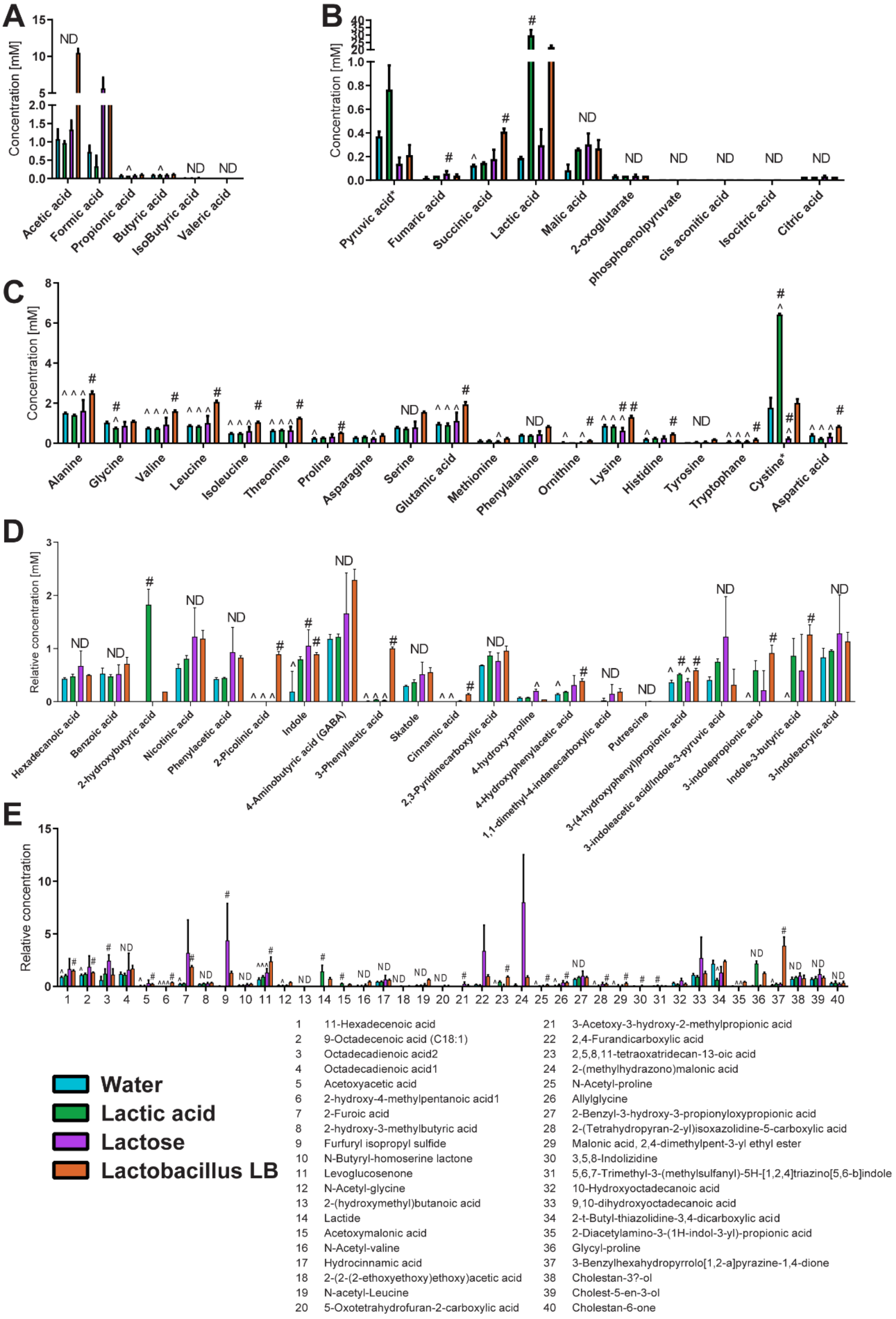

Figure S2. Annotated metabolite levels before the 24-hour faecal fermentation. Vessels were supplemented with water (blue), lactic acid (green), lactose (purple), or Lactobacillus LB (orange).

(A) SCFA. Before the start of the fermentation, there were low levels of individual SCFAs, and we observed no differences between the vessels in levels of acetic acid (Kruskal-Wallis test p=0.037; post-hoc Bonferroni p>0.05), isobutyric acid (Kruskal-Wallis test p=0.023; post-hoc Bonferroni p>0.05), and valeric acid (Kruskal-Wallis test p=1.000). In the Lactobacillus LB vessels, we detected slightly higher levels of butyric acid (Kruskal-Wallis test p=0.030; post-hoc Bonferroni p=0.050) and propionic acid (Kruskal-Wallis test p=0.026; post-hoc Bonferroni p=0.016) compared to lactic acid vessels at the start of the fermentation, but no difference compared to water or lactose vessels (Kruskal-Wallis test p<0.05; post-hoc Bonferroni p>0.05). Before fermentation, levels of formic acid differed only between lactic acid and lactose vessels (Kruskal-Wallis test p=0.012; post-hoc Bonferroni p=0.013). Levels of isovaleric acid, hexanoic acid, heptanoic acid, and valeric acid in all samples before fermentation were below the limit of detection (LOD).

(B) Citric acid cycle compounds. Before fermentation, elevated levels of succinic acid were detected in the Lactobacillus LB vessels (Kruskal-Wallis test p=0.018, post-hoc Bonferroni p=0.010) and elevated fumaric acid was also detected in lactose vessels (F(3,9)=5.599, p=0.019; post-hoc Bonferroni p=0.017), both compared to water. As expected before fermentation, levels of lactic acid were higher in lactic acid vessels (Kruskal-Wallis test p=0.010, post-hoc Bonferroni p=0.008) compared to water. At this time, lactic acid levels were higher in Lactobacillus LB vessels compared to water, but did not pass the multiple comparison criteria (Kruskal-Wallis test p=0.010, pairwise comparison post-hoc Bonferroni p=0.028, multiple comparisons adjusted post-hoc Bonferroni p=0.171). Before fermentation, a higher pyruvate level was detected in lactic acid compared to lactose vessels (Kruskal-Wallis test p=0.013, post-hoc Bonferroni p=0.019). There were no differences in the levels of the remaining six TCA cycle compounds between the vessels (Kruskal-Wallis test p>0.05).

(C) Amino acid levels. Before fermentation, 12 of 19 tested amino acids were found to be present at levels that were elevated in the Lactobacillus LB vessels, in comparison to water vessels. In particular, before fermentation tryptophan levels were higher in Lactobacillus LB vessels compared to all other vessels (F(3,9)=17.431, p<0.0005; post-hoc Bonferroni p≤0.006).

(D) Other identified compounds. Before fermentation, among the remaining 21 identified, only 8 of these were found to be present at elevated levels in the Lactobacillus LB vessels compared to water vessels.

(E) Annotated compounds. Before fermentation, 12 out of 40 annotated compounds were found to be present at elevated levels in the Lactobacillus LB vessels compared to water vessels.

The experiment was performed in triplicate or quadruplicate. # indicates significant change compared to the water vessel. ^ indicates significant change compared to Lactobacillus LB vessel. ND indicates no statistical difference between any of the vessels. * indicates relative measurement.

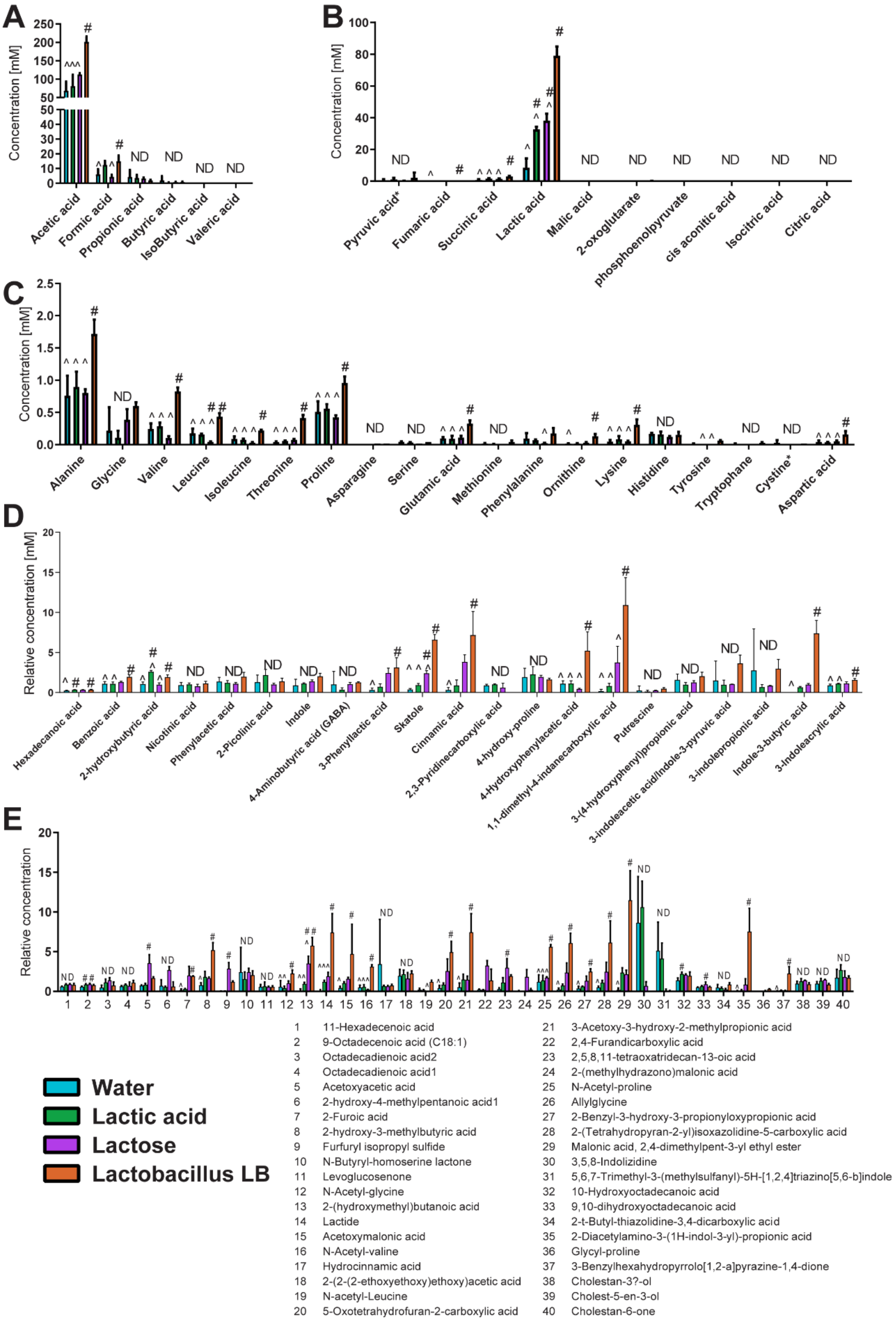

Figure S3. Annotated metabolite levels after the 24-hour faecal fermentation. Vessels were supplemented with water (blue), lactic acid (green), lactose (purple), or Lactobacillus LB (orange).

(A) SCFA. After 24 h fermentation, we observed elevated levels of acetic acid in Lactobacillus LB vessels compared to all other vessels (Fig. S3A; $F(3,9)=24.246$, $p<0.0005$; post-hoc Bonferroni $p\leq0.004$). Additionally, after 24 h fermentation formic acid levels were significantly higher in Lactobacillus LB vessels compared to water and lactose vessels ($F(3,9)=8.155$, $p=0.006$; post-hoc Bonferroni $p=0.029$ and $p=0.015$, respectively). After fermentation, there were no significant differences between vessels in propionic acid ($F(3,9)=0.382$, $p=0.769$), butyric acid (Kruskal-Wallis test $p=0.401$), isobutyric acid (Kruskal-Wallis test $p=0.094$), and valeric acid (Kruskal-Wallis test $p=0.205$) levels. Levels of isovaleric acid, hexanoic acid and heptanoic acid in all samples were below the limit of detection (LOD).

(B) Citric acid cycle compounds. After the 24h fermentation, levels of fumaric acid (Kruskal-Wallis test $p=0.025$, post-hoc Bonferroni $p=0.035$), succinic acid ($F(3,9)=22.239$, $p<0.0005$; post-hoc Bonferroni $p<0.0005$), and lactic acid ($F(3,9)=120.294$, $p<0.0005$; post-hoc Bonferroni $p<0.0005$) were elevated in Lactobacillus LB vessels compared to water. After fermentation, levels of lactic acid were also elevated in lactic acid ($F(3,9)=120.294$, $p<0.0005$; post-hoc Bonferroni $p=0.001$) and lactose ($F(3,9)=120.294$, $p<0.0005$; post-hoc Bonferroni $p<0.0005$) vessels compared to water but not to the levels found in Lactobacillus LB vessels (post-hoc Bonferroni $p<0.0005$ for both). There were no differences in the levels of the remaining six TCA cycle compounds between the vessels (Kruskal-Wallis test $p>0.05$).

(C) Amino acid levels. After fermentation, ten of 19 tested amino acids were found to be present at higher levels in the Lactobacillus LB vessels, in comparison to water vessels.

(D) Other identified compounds. After fermentation, among the remaining 21 identified, 10 of these were found to be present at elevated levels in the Lactobacillus LB vessels compared to water vessels.

(E) Annotated compounds. After fermentation, 16 out of 40 annotated compounds were found to be present at elevated levels in the Lactobacillus LB vessels compared to water vessels.

The experiment was performed in triplicate or quadruplicate. # indicates significant change compared to the water vessel. ^ indicates significant change compared to Lactobacillus LB vessel. ND indicates no statistical difference between any of the vessels. * indicates relative measurement.

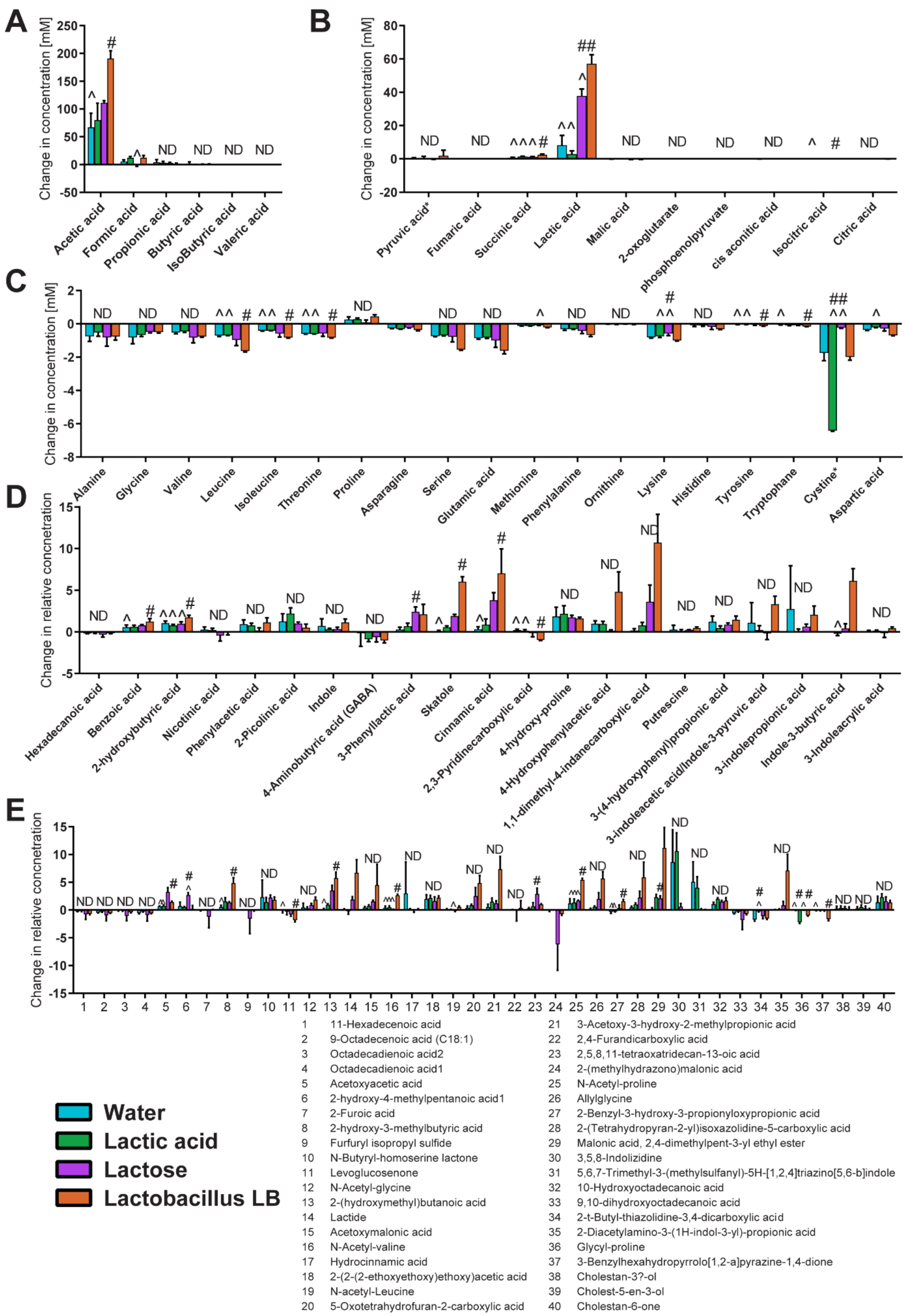

Figure S4. Change in annotated metabolite levels during the 24-hour faecal fermentation. Vessels were supplemented with water (blue), lactic acid (green), lactose (purple), or

Lactobacillus LB (orange). The experiment was performed in triplicate or quadruplicate. # indicates significant change compared to the water vessel. ^ indicates significant change compared to Lactobacillus LB vessel. ND indicates no statistical difference between any of the vessels. * indicates relative measurement.
